# South African Ebola diagnostic response in Sierra Leone: A modular high biosafety field laboratory

**DOI:** 10.1371/journal.pntd.0005665

**Published:** 2017-06-19

**Authors:** Janusz T. Paweska, Petrus Jansen van Vuren, Gunther H. Meier, Chantel le Roux, Ousman S. Conteh, Alan Kemp, Cardia Fourie, Prabha Naidoo, Serisha Naicker, Phumza Ohaebosim, Nadia Storm, Orienka Hellferscee, Lisa K. Ming Sun, Busisiwe Mogodi, Nishi Prabdial-Sing, Desiree du Plessis, Deidre Greyling, Shayne Loubser, Mark Goosen, Stewart D. McCulloch, Terence P. Scott, Alexandra Moerdyk, Wesley Dlamini, Kelfala Konneh, Idrissa L. Kamara, Dauda Sowa, Samuel Sorie, Brima Kargbo, Shabir A. Madhi

**Affiliations:** 1 National Institute for Communicable Diseases, National Health Laboratory Service, Sandringham, South Africa; 2 School of Pathology, University of the Witwatersrand, Johannesburg, South Africa; 3 Department of Microbiology and Plant Pathology, University of Pretoria, Pretoria, South Africa; 4 National Reference Laboratory, Ministry of Health and Sanitation, Freetown, Sierra Leone; University of Geneva Hospitals, SWITZERLAND

## Abstract

**Background:**

In August 2014, the National Institute for Communicable Diseases (NICD) in South Africa established a modular high-biosafety field Ebola diagnostic laboratory (SA FEDL) near Freetown, Sierra Leone in response to the rapidly increasing number of Ebola virus disease (EVD) cases.

**Methods and findings:**

The SA FEDL operated in the Western Area of Sierra Leone, which remained a “hotspot” of the EVD epidemic for months. The FEDL was the only diagnostic capacity available to respond to the overwhelming demand for rapid EVD laboratory diagnosis for several weeks in the initial stages of the EVD crisis in the capital of Sierra Leone. Furthermore, the NICD set out to establish local capacity amongst Sierra Leonean nationals in all aspects of the FEDL functions from the outset. This led to the successful hand-over of the FEDL to the Sierra Leone Ministry of Health and Sanitation in March 2015. Between 25 August 2014 and 22 June 2016, the laboratory tested 11,250 specimens mostly from the Western Urban and Western Rural regions of Sierra Leone, of which 2,379 (21.14%) tested positive for Ebola virus RNA.

**Conclusions:**

The bio-safety standards and the portability of the SA FEDL, offered a cost-effective and practical alternative for the rapid deployment of a field-operated high biocontainment facility. The SA FEDL teams demonstrated that it is highly beneficial to train the national staff in the course of formidable disease outbreak and accomplished their full integration into all operational and diagnostic aspects of the laboratory. This initiative contributed to the international efforts in bringing the EVD outbreak under control in Sierra Leone, as well as capacitating local African scientists and technologists to respond to diagnostic needs that might be required in future outbreaks of highly contagious pathogens.

## Author summary

In response to Ebola virus disease outbreak in West Africa, the National Institute for Communicable Diseases in South Africa established a modular high-biosafety field Ebola diagnostic laboratory (FEDL) near Freetown, Sierra Leone. This was the sole diagnostic capacity available to respond to the overwhelming demand for Ebola diagnosis for several weeks in the Western Area of Sierra Leone. The deployment of the FEDL capacity contributed to the overall international efforts in bringing the Ebola outbreak in West Africa under control.

## Introduction

Ebola virus (EBOV) was previously only identified to cause outbreaks of Ebola virus disease (EVD) in central equatorial Africa [1], but emerged in West Africa in late 2013 in Guinea [2], and spread into Liberia in March, Sierra Leone in May, and Nigeria in late July 2014 [3]. The EVD outbreak was declared by the World Health Organization (WHO) as a public health emergency of international concern (PHEIC) on 8 August 2014 [4]. The United Nations (UN) Security Council recognised the outbreak as a threat to peace and security on 19^th^ September 2014 [5], and consequently the UN Mission for Ebola Emergency Response (UNMEER) was deployed to combat the Ebola crisis in West Africa [6]. The index case is thought to have become infected late in December 2013 in a village located in the forest region of south-east Guinea [2, 7]. EVD spread unrecognised from its unexpected source in Guinea to the neighbouring countries [8] to become the largest outbreak in the recorded history of the disease [9, 10]. The PHEIC related to EVD in West Africa was formally lifted on 29 March 2016 [11]. A total of 28,616 confirmed, probable and suspected cases were reported in Guinea, Liberia and Sierra Leone, with 11,310 deaths [12]. Confirmed EVD cases among health care workers (HCWs) amounted to 881 of which 513 (58.2%) were fatal [13].

The emergence of EVD in West Africa had a severe impact on public health, social life, national education programmes, agriculture, transportation, domestic and international markets [14]. The high EVD death toll amongst HCWs had impaired the already weak health system in Guinea, Sierra Leone, and Liberia, and might have a long term and severe effect on maternal, infant and under-5 year mortality [15]. A scarcity of resources in testing for EBOV infection left patients stranded in isolation wards for days with little to no management. The establishment of rapid and more widely accessible diagnostic capacities in the West African countries affected by the EVD epidemic was identified as one of the most urgent priorities to prevent the further escalation of the EBOV-related health and humanitarian crisis [10, 16].

The first EVD cases in Sierra Leone were reported in May 2014 in the Kailahun District that shares its borders with Guinea and Liberia. The disease subsequently spread to the neighbouring Kenema District in June 2014. Between 23 May and 30 August 2014, the two districts had the highest number of EVD confirmed cases, with the incidence rates during this time of 3.8 and 7.0 per 100 000 population per week, respectively [17]. Laboratory diagnosis of the first EVD cases in Sierra Leone was made on 25 May by the Lassa diagnostic laboratory at the Kenema Government Hospital (KGH) [18]. KGH was the only facility in the country that could provide care to EVD patients at the onset of the outbreak [19]. The first cases were likely exposed to EBOV when attending the funeral of a traditional healer who had been treating Ebola victims in neighbouring Guinea [3, 18]. Due to severe staff losses, increasing numbers of EVD patients, shortages in the medical supplies, compounded by hostile situations and striking of the remaining nurses and laboratory technicians, operations of both the KGH and the Lassa diagnostic laboratory were difficult to continue and needed urgent assistance. Aid was eventually provided by Médecins Sans Frontières/Doctors Without Borders (MSF), WHO and other international partners [18, 19]. The cluster of EVD cases involving HCWs in the Kenema District was one of the largest ever reported in the history of the disease [19]. Of the eight laboratory technicians, only one survived the EVD outbreak [20]. By late July 2014, EVD cases were detected in the capital of Sierra Leone, Freetown [3, 10]. The fear that the deadly virus would spread to the congested, impoverished, post-conflict West African urban areas had become reality. Since the introduction of EBOV into Sierra Leone, new EVD cases were reported daily in increasing numbers in nearly all provinces, but particularly in the Western Urban and Western Rural regions of Sierra Leone. During the period May 23 to October 31, 2014, there were 3,854 laboratory-confirmed cases of EVD reported in Sierra Leone, including 199 cases in HCWs. The confirmed EVD incidence was 103-fold higher in HCWs than that in the general population in Sierra Leone [21]. The weak health system, lack of experience with management of EVD, poor community awareness, and highly deficient laboratory diagnostic capacity further contributed to the spread of the disease [10, 16, 22, 23].

In response to the public health emergency caused by the EVD epidemic, the National Institute for Communicable Diseases (NICD) established a modular high biosafety field Ebola diagnostic laboratory (FEDL) within the compound of the Lakka Tuberculosis (TB) Hospital located on the outskirts of Freetown in the second half of August 2014.

This paper describes the deployment, organisation, diagnostic and biosafety procedures utilised by the South African (SA) FEDL in Sierra Leone, the training of Sierra Leonean national staff during the EVD outbreak, laboratory results, and the hand-over of the SA FEDL to the Ministry of Health and Sanitation (SL MoHS) as a part of the SA EVD diagnostic capacity building initiative in West Africa.

## Methods

### Ethical statement

We did not seek institutional review board approval for data collection in this study because data were collected as part of routine case management under an emergency response mandate from the government of Sierra Leone. As a part of routine practise, patients orally agreed to be tested for Ebola virus infection. Permission to carry out post-outbreak studies on samples transported from Sierra Leone to BSL4 facility in South Africa was given by the Government of Sierra Leone Office of the Sierra Leone Ethics and Scientific Review Committee, Directorate of Training and Research, Ministry of Health and Sanitation.

### Pre-deployment training and deployment

To support the SA FEDL initiative in Sierra Leone, South African scientists and technologists throughout the NICD and the University of Pretoria were voluntarily mobilised. They undertook intensive in-house pre-deployment training in the safety and diagnostic procedures of the FEDL and a further 2–4 days on-site training by the preceding team upon arrival in Sierra Leone. The SA FEDL staff members completed the UN basic and advanced field safety and security programme training and were all deployed through the WHO Global Outbreak Alert and Response Network recruitment system.

### Air transport of the SA FEDL staff to Freetown, Sierra Leone

Travel restrictions leading to the discontinuation of commercial flights to West African countries affected by the EBOV epidemic presented difficulties in the rotation of SA FEDL teams and shipping of laboratory supplies to Freetown. The UN World Food Programme established the Humanitarian Air Service (UNHAS) to augment the capacity of humanitarian efforts under UNMEER. With the assistance of the SA National Department of Health, most of SA FEDL teams were transported using commercial flights from Johannesburg via Dakar (Senegal) or Accra (Ghana) combined with UNHAS flights to Freetown.

### SA NICD FEDL operation and biosafety manual

The infrastructural and operational capacity of the SA FEDL was designed to primarily conduct molecular EVD and Lassa fever laboratory testing and to ensure adequate safety procedures for working with BSL-4 pathogens under field conditions in a poorly resourced country. Originally, the diagnostic capacity of the laboratory was designed to process and test a maximum of 58 specimens per day.

### Establishment of the SA FEDL

The first SA team consisted of four NICD staff members experienced in laboratory diagnostics of viral haemorrhagic fevers (VHFs), and the construction and technical management of biocontainment facilities. This team was tasked with the selection of an appropriate site for the deployment of the SA FEDL and establishing its full operation. The team arrived in Freetown, on 17 August 2014 and the following day visited the WHO Country Office where it was introduced to the National Coordination Team of the Ebola Epidemic Response. The same day, the team visited the headquarters of the UN Development Programme in Freetown to obtain country security briefing by the UN Department of Safety and Security. On 18 August the team visited the Lakka Tuberculosis (TB) Hospital in Freetown where potential operating sites for the SA FEDL were inspected. The next day, the decision was made to deploy the SA FEDL to the campus of the Lakka TB Hospital, in a vacant building planned to be used in the future as a TB reference laboratory. On 19 August, the team inspected the interior structure and the layout of the building and started preparations for the establishment of the SA FEDL.

On 20 August, the first shipment containing the negative pressure biological containment system (IsoArk^®^, Beth-el Zikhron Yaaqov Industries Ltd., Zikhron Yaaqov, Israel) arrived from South Africa and the same day it was assembled in a designated room for “hot” processing of specimens. The second shipment containing laboratory equipment, a negative pressurised glovebox, rapid containment kits, generator, uninterrupted power supplies (UPS), reagents, laboratory consumables, personal protective equipment, and other SA FEDL operation supplies arrived on 22 August. On 24 August, mock EVD testing, using synthetic RNA controls, was conducted to evaluate the work flow and the performance of the EBOV reverse transcription polymerase chain reaction (RT-PCR) assay under field conditions and to confirm the integrity of reagents after shipping. On 25 August the SA—FEDL was declared fully operational and the testing of the first blood specimens from EVD suspected cases commenced.

### General description and structural components of FEDL

**Fig 1:**
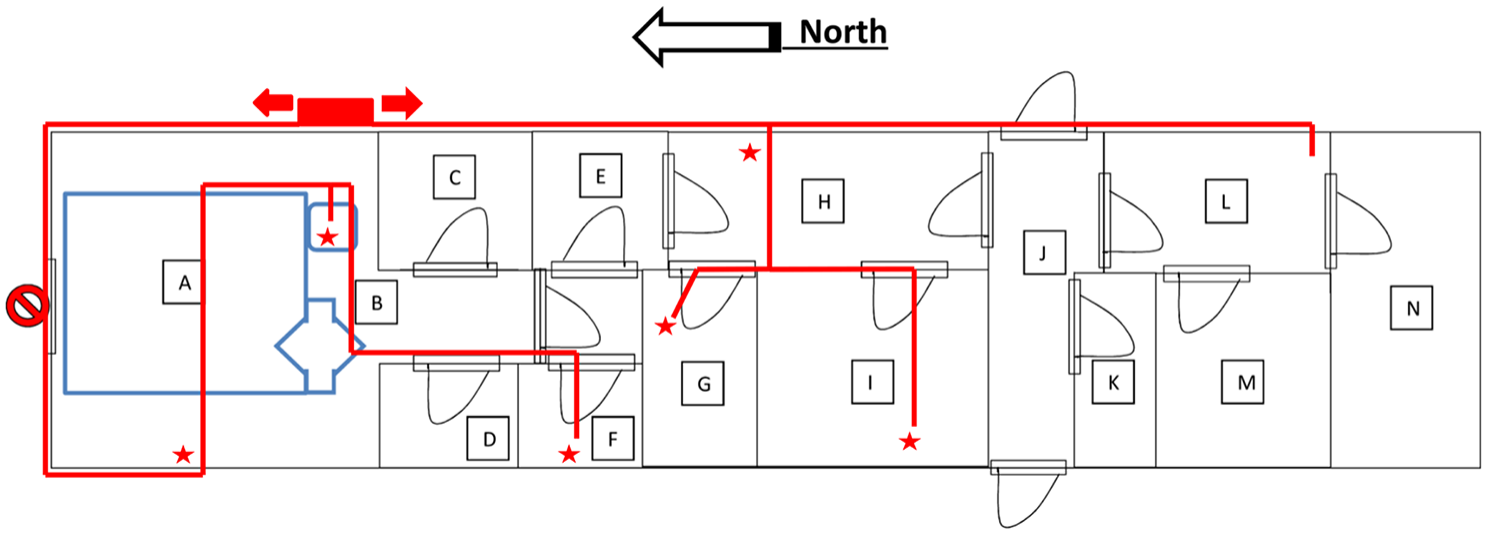
Layout of the SA FEDL in Freetown-Lakka, Sierra Leone with emergency generator and wiring to allow for rapid switch to generator mode in case of power failure. (**A**) Biocontainment negative pressure chamber (IsoArk), (**B**) Room housing biocontainment negative pressure chamber, (**C**) Donning room, (**D**) Doffing room, (**E**) Laboratory airlock area, (**F**) PCR amplification room, (**G**) PCR master mix room, (**H**) Specimens and reagents storage area, (**I**) RNA extraction room, (**J**) Facility entrance, (**K**) Toilet, (**L**) Office 1, (**M**) Office 2, (**N**) Office 3. Petrol generator (5.5 kVa) placement indicated by the red rectangle, distribution of extension cords are indicated with red lines, and emergency connection points by red stars.

The FEDL was located on the premises of the Lakka TB Hospital compound, Peninsula Road, Lakka near Freetown, in a building consisting of brick structure, steel roofing, lockable windows and three lockable entrances. All windows and doors were fitted with burglar bars. Air conditioner units were located in most of the rooms of the building. Two emergency electricity generators of different output capacities were used. The main back-up 60 kVA diesel-powered generator was routed to the main distribution board to supply electricity to the entire building. The second 5.5 kVA petrol generator was fed to the building by means of electrical extension cords and only supplied power to essential laboratory and storage equipment such as biocontainment devices, real time PCR instruments, refrigerators and freezers. In addition, essential laboratory equipment was connected to UPS to prevent power loss during operation and to serve as voltage stabilisers. The lay-out of the SA FEDL is given in Fig 1. Procedures for centrifugation, aliquoting and inactivation of specimens as well as the addition of positive control RNA and the RT-PCR amplification of RNA templates were carried out in designated rooms that were separated from the PCR master mix preparation room, RNA extraction room and storage area by a laboratory airlock area (Fig 1).

### Decontamination procedures

Chlorine disinfectant solution (Medisure) at a concentration of 0.5% (5000 ppm) was used for the decontamination of pipette tips that came into contact with clinical specimens, primary specimen containers, the exterior of sample tubes containing plasma/lysis buffer in ziplock bags for their transfer out of biocontainment, and for the decontamination of the glovebox working area during and after “hot” processing. A lower concentration of 0.05% chlorine (500 ppm) was used for all other decontamination procedures, including but not limited to decontamination of operators PPE when exiting the IsoArk, decontamination of specimen submission forms, secondary containers, work benches, and floors. Work benches in the RNA extraction and PCR master mix rooms, rapid containment kits and class II biosafety cabinet work surfaces were decontaminated daily with 0.05% chlorine disinfectant solution followed by wipe down with 70% ethanol. At the end of each day, the ultra-violet (UV) germicidal lamp fitted to the biosafety cabinet was operated for 15 minutes, while portable UV germicidal lamps were operated for 20 minutes in the RNA extraction and PCR master mix rooms.

### Receiving of specimens

Staff receiving specimens wore PPE that included, a surgical gown, gloves, a N95 mask and eye protection. The outer boxes/specimen containers, as well as case investigation forms were first placed into plastic biohazard bags and then taken to the airlock of the IsoArk for decontamination by mist spray with chlorine disinfectant. Specimens were then either moved to the temporary storage fridge for later processing or taken into IsoArk biocontainment chamber for immediate processing. Specimens were cross-matched with the information provided in the case investigation forms. A unique numerical laboratory identification number was assigned to each specimen. Information on specimens accepted for testing was recorded in the specimen register and included a laboratory submission number, a patient name and surname, age, sex, reference number and name of referral facility. More detailed information was collected and electronically recorded from case investigation forms by the WHO employed data capture clerk, using the reporting template provided by the SL MoHS. Reception of specimens was refused or subjected to storage until specimen submission forms could be obtained or if the required patient history could be provided by other confirmable means.

### Patient data

Patient data captured electronically were copied into the Ebola master database with the corresponding FEDL reference laboratory submission number and the sample RT-PCR cycle threshold (Ct) value. The leader of each FEDL rotation team was responsible for ensuring that the master database was safely maintained and that the data and confidentiality of results was safeguarded. Results were mailed electronically to authorised email addresses using the reporting template supplied by the SL MoHS and WHO. Urgent results were communicated directly to attending healthcare workers by telephone, while reporting of results to family or community members by FEDL staff was strictly forbidden.

### Safety and containment measures

**Fig 2:**
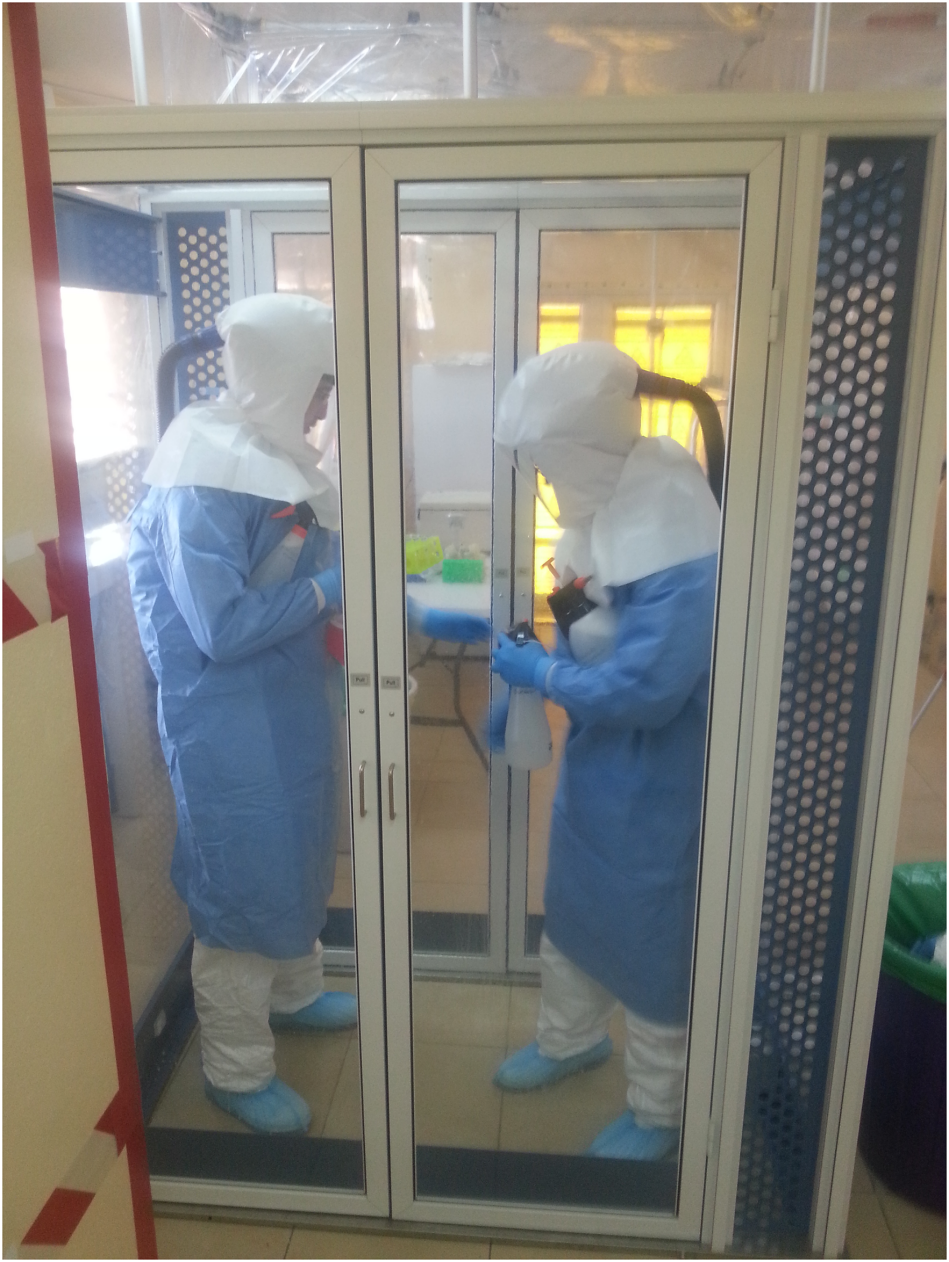
Operators dressed in BSL3 PPE (scrubs, Tyvek suits, surgical gown, double pair surgical gloves, gumboots, overshoes and PAPR with full face hood) entering the IsoArk main chamber through the airlock.

Protection of staff from direct or indirect contact with infectious substances present in clinical samples or in droplets that could be formed during handling and processing of diagnostic specimens was achieved using PPE (Fig 2), including a Powered Air Purifying Respirator (PAPR) coupled to a full-face hood. The full set of PPE for “hot” work in the IsoArk for one operator included: washable socks; single use overshoes; a single use scrub set (pants and shirt); a single use Tyvek coverall; reusable gumboots; two pairs of single use surgical examination gloves; one pair of single use long-cuff examination gloves; a single use fluid resistant, rear fastening folio gown and the reusable parts of the PAPR (filter-blower unit, air-hose, double shrouded full-face hood and rechargeable battery).

**Fig 3:**
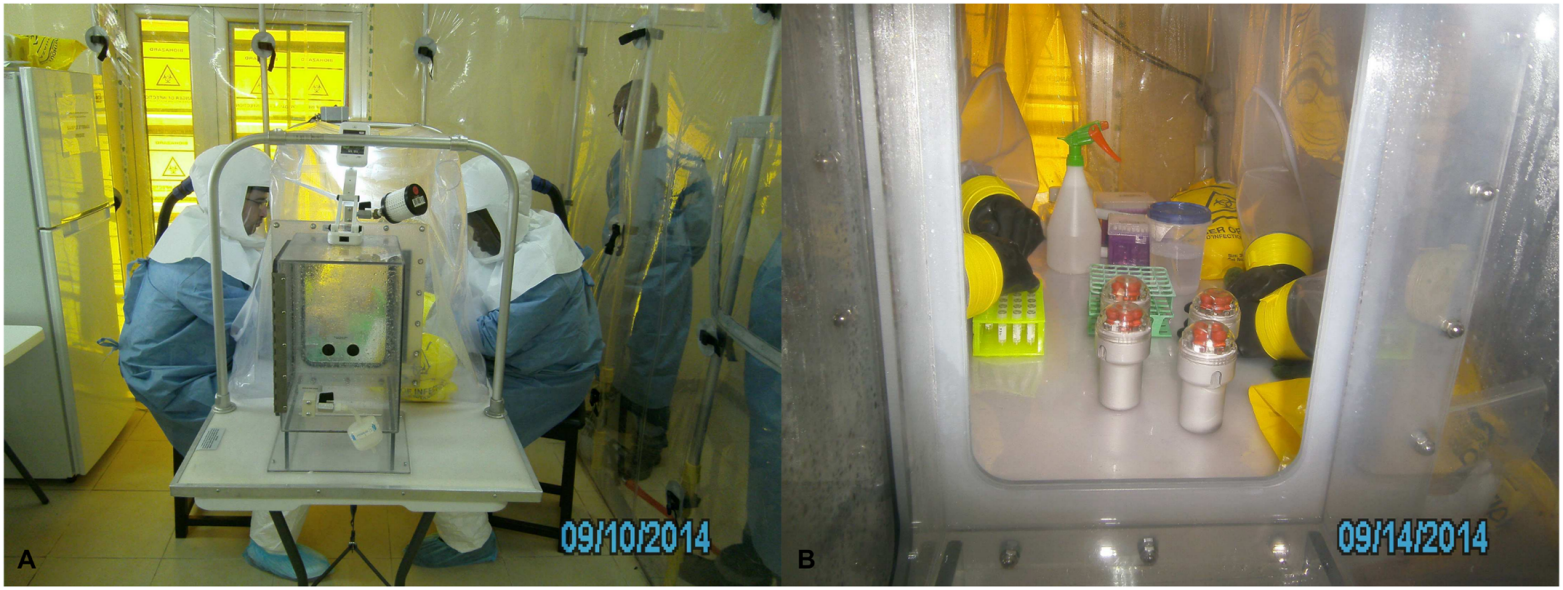
(**A**) Operators dressed in BSL3 PPE processing clinical specimens from EVD suspected cases in a glovebox located within the IsoArk biocontainment negative pressure chamber, (**B**) Blood tubes in centrifuge adapters with safety caps passed into the main chamber of the glovebox for “hot” inactivation and aliquoting for long-term storage.

The IsoArk (Figs 2 and 3) features a high air change rate, low noise level, and adjustable airflow rate up to 2,200 m^3^/hr. The main components of IsoArk included: an airlock, a main chamber, and an air filtration system. When entering the airlock, an electro-optical eye sensor automatically switched the filtration system to a high flush mode that increased the airflow through the airlock. This reduced the waiting time for a complete air change in the airlock and ensured that the negative pressure was maintained even when entering or exiting the main chamber. Electrical cables were passed into the isolated area through specialised utility sleeves. The integrated air Filtration System FA 2000 combined a highly efficient HEPA-filter with a UV-radiation source. The glovebox used for primary containment of specimens (Fig 3) features a negatively pressurised vinyl box that allows safe processing of biohazardous material and a solid perspex pass-box that enables safe passage of materials into and out of the containment area.

PPE worn by staff exiting the containment area was decontaminated by two rounds of mist-spray using a pressurised container with spray nozzle containing 0.05% chlorine disinfectant. The first mist spray was performed within the IsoArk biocontainment. After the removal and disposal of overshoe coverings and the first pair of gloves into a biological waste bag, a second mist-pray was performed inside the airlock. After allowing 3 minutes of contact time with the disinfectant solution, operators were permitted to leave the airlock and complete PPE doffing. Procedures in the “hot” room followed by exit from IsoArk are additionally illustrated in supplementary video material (S1 Video).

### Manual viral RNA extraction

Manual RNA extraction was carried out using the QIAmp viral RNA kit (Qiagen, Hilden, Germany) according to the manufacturer’s instructions. Clotted or whole blood in vacutainer tubes were centrifuged, using safety buckets, inside the IsoArk for 3 minutes at 1,500 x g to separate serum/plasma from cellular content. Following centrifugation, the sealed buckets containing the blood tubes and swabs were placed inside the glovebox. Serum/plasma or swab medium was transferred to the pre-labelled tube containing AVL-carrier RNA buffer. Remaining plasma was transferred into an empty cryotube for long-term storage. The cryotubes containing processed specimens and biohazard bags containing the original blood tubes were mist-sprayed before transfer from the glovebox into the main chamber of the IsoArk. To ensure effective and rapid inactivation of EBOV in specimens before removal from biocontainment, a volume of 560 μl molecular grade absolute ethanol was added to each sample/AVL tube [24, 25]. Tubes containing inactivated specimens were then placed into an airtight ziplock bag filled with chlorine disinfectant. The ziplock bag exterior was mist-sprayed and moved to the airlock of the IsoArk, and after 10 minutes contact time for inactivation was transferred to the RNA extraction room to finalize extraction. Extracted RNA specimens were transferred to the 4°C fridge until later addition of the PCR mastermix. Used blood tubes or tubes containing buccal swabs were filled with chlorine disinfectant within the glovebox, closed, and transferred from the glovebox to the IsoArk biocontainment area, where they were placed in a biological waste bag for incineration.

### Automated viral RNA extraction

Automated RNA extraction was carried out according to the manufacturer’s instructions (MagMax96, ThermoFisher Scientific, Waltham MA, USA) using MagMax Express-96 Deep Well magnetic particle extractor (Life Technologies, New York, USA). Clotted blood was centrifuged as described for processing of blood specimens for manual extraction. Vacutainer tubes containing whole blood samples (EDTA or heparin blood) were not centrifuged. Serum, whole blood or swab medium was transferred into the pre-labelled cryotube containing MagMax lysis buffer and carrier RNA. The remaining volume of processed serum, or buccal swab medium was transferred to a cryotube for long-term storage. Vacutainer tubes containing the remaining whole blood were not centrifuged but were temporarily stored at 4°C in case of the need for retesting. If not required for retesting, the next day the remaining blood was centrifuged and plasma aliquoted for long-term storage. Extracted RNA was aliquoted into pre-labelled tubes and stored at 4°C until addition to the PCR master mix.

### Comparison of automated versus manual extraction

To evaluate the potential impact of automated versus manual RNA extraction on the diagnostic performance of EBOV L gene TaqMan assay, EBOV RNA was extracted from 72 sera of EVD suspected cases using both extraction methods, and subjected to comparative analysis.

### Polymerase (L) gene TaqMan real-time RT-PCR

The real time RT-PCR protocol for the detection of EBOV RNA targeting the polymerase (L) gene was carried out as previously described [26]. The assay was run using real-time PCR instrument (SmartCycler, Cepheid, Sunnyvale, CA, USA). The manufacturer’s protocol was followed when preparing the RT-PCR master mixes (Qiagen One-Step RT-PCR kit, Qiagen, Hilden, Germany).

### Internal quality control and interpretation of results

The monitoring of quality performance of the EBOV L gene TaqMan assay involved testing of internal RNA controls having pre-determined Ct values. The L gene TaqMan assay run was considered valid when the following internal quality control (IQC) acceptance criteria were met: (1) the positive RNA control had a Ct value < 35 and fell within the predetermined upper (UCL) and lower (LCL) IQC limits (2) the positive extraction control had a Ct value < 35 and fell within the predetermined UCL and LCL IQC limits, (3) no amplification was detected in the negative control, and (4) no amplification was detected in the negative extraction control.

When the IQC criteria for the PCR run were met, the results in the test sample were interpreted as follows: (1) positive result when Ct value was ≤40, and (2) negative results when no amplification was detected or the Ct value was > 40. To exclude possible low level of cross-contaminations, specimens yielding high Ct values, from 35 to 40, were retested using re-extracted RNA. Clinicians were advised that patients who tested negative and with Ct values of 35 to 40 should be re-bleed and tested again.

The SA FEDL took part in two external quality assessment runs conducted by WHO Country Office Sierra Leone, the Centers for Disease Control and Prevention (CDC) Atlanta, WHO and the European Network for Diagnostics of Imported Viral Diseases [27].

### Bio-waste management

Laboratory generated bio-waste was burned in 210 litre capacity oil drums re-designed for field incineration using diesel fuel as ignition source.

## Results

### Rotation of SA FEDL staff

Initially, the operation of the SA FEDL was planned for 2–3 teams and for a maximum of 3 months duration. This plan had to be revised following the ground experience of the first SA FEDL team. The lack of effective control of the EVD outbreak in the second half of 2014 prompted the need for drastic upscaling of the EVD diagnostic capacity in Sierra Leone Preparations were then made for the deployment of up to ten SA teams. However, this was eventually not required due to the success of outbreak response measures that resulted in controlling the EVD crisis by the end of January 2015. In addition, the training of national staff enabled the handover of the SA FEDL to SL MoHS in March 2015.

Eight SA teams each consisting of 2–5 members, were deployed from 17 August 2014 to 25 March 2015. Each team was deployed for 5–7 weeks, with 2–4 days overlap with the outgoing team. Each team worked 12–16 hours a day, 7 days a week, and usually no days off. On most days, RNA extractions and RT-PCR were performed both in the morning and afternoon.

### Training of local staff

After two weeks of operation in Freetown, FEDL staff undertook intensive training of five Sierra Leonean staff in all aspects of the FEDL logistic and diagnostic operations. The Sierra Leonean nationals were gradually incorporated into all routine functions of the FEDL and became invaluable in its long-term and sustainable operation. Of the five Sierra Leonean nationals trained, two had BSc degree and two had MSc degree in biology, one had Diploma in Paramedic Sciences.

### Laboratory results

**Fig 4:**
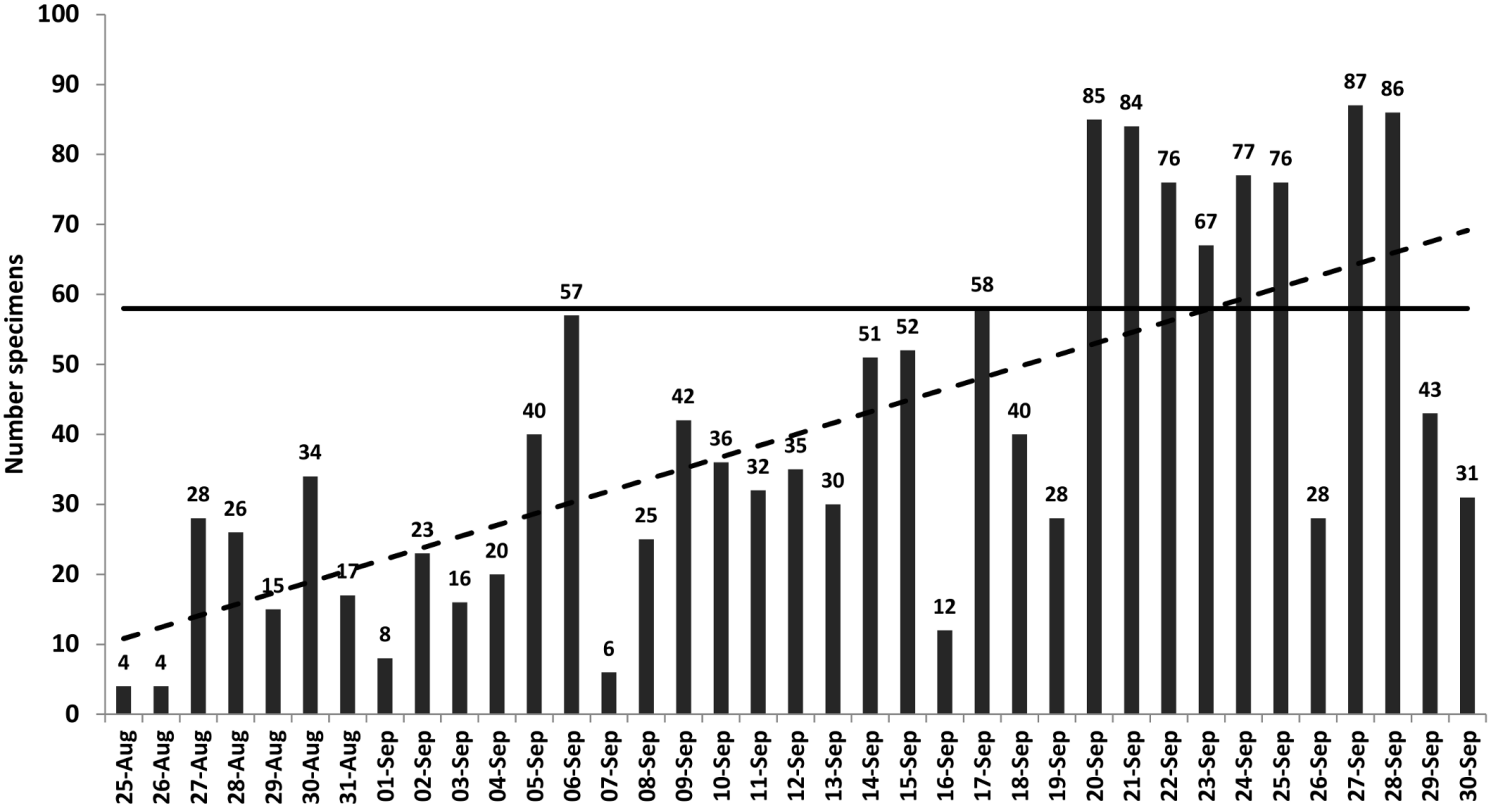
Number of specimens from EVD suspected cases in Sierra Leone tested daily (total blood and buccal swabs) by SA FEDL during the first weeks of operation, 25 August—30 Sept 2014. Column = Number of specimens tested daily; Dotted line = Trend line of a number of specimens tested; Solid line = Maximum testing capacity of 58 specimens per day.

Specimens from suspected EVD cases were submitted to the FEDL throughout the day, sometimes until late at night. During the first weeks of operation, specimens were submitted from several regions of Sierra Leone. Specimens received from October 2014 onwards mostly originated from Freetown health care facilities, including the Emergency Ebola Treatment Centre, Connaught Hospital, 34 Military Hospital, Macauley Street Clinic, Hastings Ebola Treatment Centre, Princess Christian Hospital, Lumley Governmental Hospital, and Ola During Children’s Hospital. The EBOV L gene TaqMan assay results were usually issued twice a day. Submission of specimens rapidly increased during the first weeks of operation and exceeded the maximum testing capacity of 58 specimens per day on several occasions during the last two weeks of September 2014 (Fig 4). The week of 14–20 September 2014 included a 3-day National House-to-House Campaign to enhance active EVD surveillance [17] and resulted in increased demand for laboratory confirmation of EVD suspected cases.

**Fig 5:**
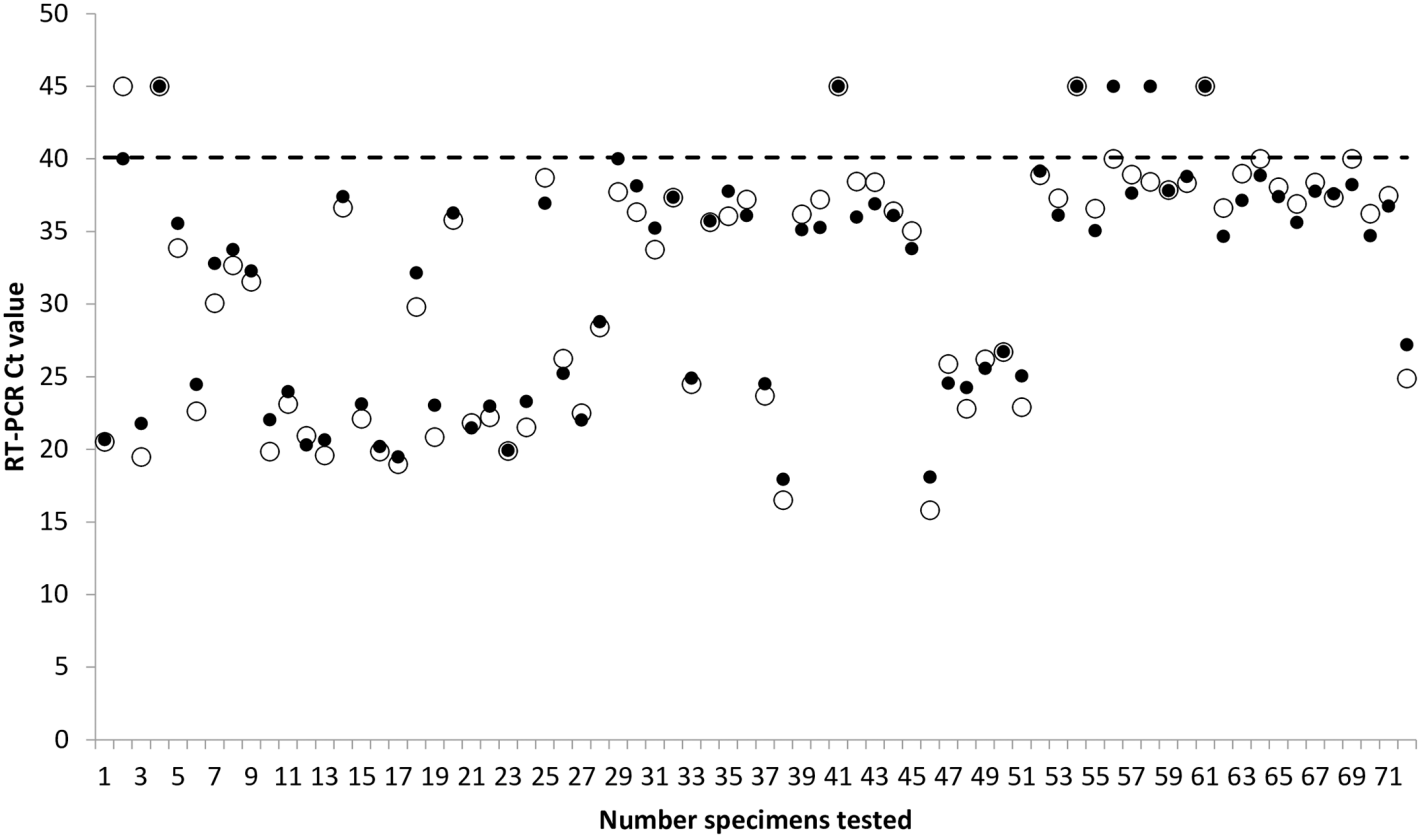
Results of EBOV L-gene RT-PCR in sera subjected to RNA manual (black dots) and automated (circles) extraction.

The increasing demand for EVD diagnosis necessitated improvement of the technical laboratory capacity. This was achieved by the use of the MagMAX^™^ 96 Express automated nucleic acid extractor. In a comparison study, the mean Ct value of 72 sera subjected to manual and automated extraction was 31.9 ±7.9 standard deviations (SD) (95% confidence interval [CI], 30.0–33.7) and 31.6±8.3 SD (95% CI, 29.7–33.6), respectively. The Wilcoxon matched-pairs signed-ranks test gave the two-tailed P value of 0.2194, indicating no significant differences between the two extraction methods used. When using the cut-off of ≤40 Ct value for the EBOV L gene TaqMan assay, of 67 positive specimens after automated extraction, 65 (97%) were also positive and two were negative after manual extraction. Of 5 negative specimens after automated extraction, 4 (80%) were also negative, and one was positive after manual extraction. The observed discrepant results were noted only in samples with high Ct values ranging from 38.42 to 40 (Fig 5).

Between 25 August 2014 and 22 June 2016, the laboratory tested 11,256 specimens mostly obtained from the Western Urban and Western Rural regions of Sierra Leone, of which 2,379 (21.1%) tested positive for Ebola virus RNA. These also included post-outbreak specimens tested during the WHO-recommended increased EVD surveillance period after the WHO declared Sierra Leone free from EVD on 07 November 2015.

From 25 August to 30 September 2014 (37 days) when the SA FEDL was the sole operational Ebola diagnostic facility in Freetown, the laboratory tested 1,219 blood samples (Table 1) and 260 buccal swabs (Table 2) of which 62.2% and 38.8% were positive by RT-PCR, respectively. From 25 August 2014 to 31 March 2015, a total of 7,267 specimens were tested, of which 4,299 were blood specimens (Table 1) and 2,968 were buccal swabs (Table 2). Over this period the percentage of positive blood samples and buccal swabs drastically decreased from 62.2% to 13.2% (Table 1) and from 38.8% to 1.4% (Table 2), respectively.

**Table 1:** Ebola virus polymerase gene TaqMan real-time RT-PCR results in blood specimens.

Month of submission	Total submissions	Number blood samples positive^1^ (%positive)	Range Ct values in positive samples	Mean Ct value ± SD^2^ in positive samples	Number potentially viremic patients (%)^3^
25 Aug/Sept 2014	1219	758 (62.2)	13.76–39.49	27.05 ± 4.71	691 (91.2)
Oct 14	644	292 (45.3)	15.28–39.66	25.64 ± 5.06	262 (89.2)
Nov 14	686	308 (44.9)	14.39–39.99	26.78 ± 5.66	261 (84.4)
Dec 14	847	266 (31.4)	12.37–38.72	25.71 ± 6.25	227 (78.1)
Jan 15	550	80 (14.5)	12.82–38.15	25.33 ± 6.65	68 (85.0)
Feb 15	201	26 (12.9)	15.35–37.23	26.37 ± 7.08	20 (76.2)
March15	152	20 (13.2)	18.87–37.31	28.82 ± 5.52	16 (80.0)
April 15–22 June 16	3170	8 (0.25)^4^	19.56–39.04	28.78 ±6.14	7 (87.5)
Total	7469	1758 (23.5)	12.37–39.99	26.5 ± 5.38	1552 (88.3)

^1^ Ct value ≤40;

^2^ Standard deviation;

^3^ Ct value <33.7 [28]

^4^ During the period of April 2015 and November 2016 of the eight positive blood specimens, two tested positive in April 2015, two in June 2015, two in August 2015, and two by the end of January 2016.

**Table 2:** Ebola virus polymerase gene TaqMan real-time RT-PCR results in buccal swabs.

Month of submission	Total submissions	Number swabs positive^1^ (%positive)	Range Ct values in positive samples	Mean Ct value ± SD^2^ in positive samples
25 Aug/Sept 2014	260	101 (38.8)	19.92–39.83	27.03 ± 4.24
Oct 14	530	199 (37.5)	17.18–39.96	27.22 ± 5.17
Nov 14	728	214 (29.4)	15.82–39.44	26.84 ± 4.27
Dec 14	605	82 (13.6)	16.11–38.72	25.76 ± 4.46
Jan 15	373	12 (3.2)	18.29–38.41	26.31 ± 6.2
Feb 15	186	8 (4.3)	18.79–30.9	22.72 ± 3.9
March 15	286	4 (1.4)	26.13–39.93	31.16 ± 6.08
April 15–22 June 16	813	1 (0.12)^3^	-	-
Total	3781	621(16.4)	15.82–40.0	26.80 ± 4.68

^1^Ct value ≤40;

^2^Standard deviation;

^3^During the period of April 2015 and November 2016 only one buccal swab tested positive (Ct value of 21.35) on 25 May 2015.

From April 2015 to 22 June 2016 (after the handover of the FEDL to the SL MoHS) a total of 3,170 blood specimens (Table 1) and 813 (Table 2) buccal swabs were tested of which 8 (0.25%) and 1 (0.12%) were positive, respectively. The last two blood specimens that tested positive were sampled at the end of January 2016 (Table 1), while the last buccal swab that tested positive was sampled in May 2015 (Table 2). Irrespective of the month of submission, the mean EBOV L gene TaqMan assay Ct values for positive blood specimens were similar. In the total number of 1,758 positive blood specimens, the Ct values ranged from 12.37 to 39.99 with a mean value of 26.5 ± 5.38 (Table 1). The mean Ct values for positive buccal swabs were also comparable across the months tested. In the total number of 621 positive buccal swabs, the Ct values ranged from 15.82 to 40.0 with mean value of 26.8 ± 4.68 (Table 2). Of the total of 1,758 RT-PCR positive blood specimens, 1,552 (88.3%) were collected from potentially viraemic EBOV patients (Table 1).

Categorisation of EBOV L gene TaqMan assay positive results as indicating infectious versus non-infectious specimens was based on recently published correlation between Ct value of the L gene TaqMan assay and virus isolation results of EBOV from blood specimens [28].

**Fig 6:**
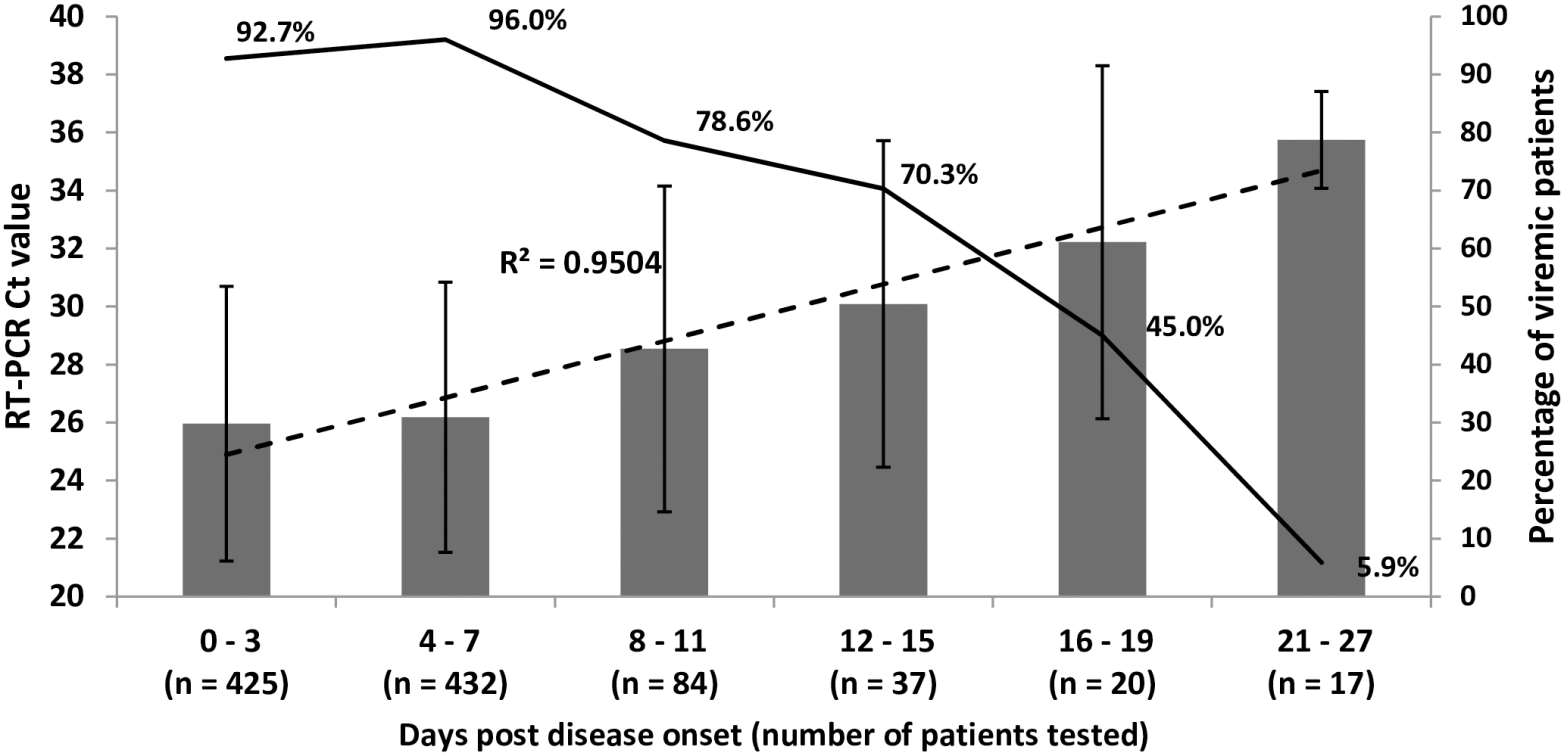
EBOV L-gene RT-PCR Ct values in sera of EVD patients tested at different days after disease onset and percentage of viremic cases. Solid column—mean ± 1SD Ct value; Dotted line—lineral correlation (R^2^) between Ct values and days post onset; Solid line—percentage of viremic patients. Ct value > 33.7 indicates non-viremic patient [28].

In 1015 patients tested RT-PCR positive for the presence of EBOV in their blood and for whom day of onset of clinical symptoms was known, there was a strong linear correlation (R^2^ 0.9504) between Ct values and the days after onset. The lowest Ct values were recorded on days 0–3 (mean 25.9 ± 4.7 SD), and the highest on days 20–27 (mean 35.7 ± 1.7 SD) after onset. The percentage of viremic patients ranged from 96 on days 4–7 (n = 432) to 5.9 on days 21–27 (n = 17, one viremic patient with CT value of 33.07 on day 21) after onset (Fig 6).

The mean time from symptoms onset to diagnosis ranged from 7.5 ± 3.8 SD days in October 2014 to 2 ± 2.7 SD in March 2015. The mean time from collection of specimens to diagnosis ranged from 2.2 ± 1.2 SD days in October 2014 to 0.7 ± 0.8 SD in March 2015. Through August 2014 to March 2015, on average 53%, 34.6%, 10%, and 2.4% results were reported <6 hours (h), <12 h, <24 h, and >24h after receiving of specimens, respectively.

Owing to the excessive workloads, assays other than EBOV RT-PCR were not performed until early March 2015. Since then malaria rapid test (SD Bioline, Standard Diagnostics, Korea) was conducted on request of the attending physician. Of 26 Ebola positive patients, one was also malaria positive (3.8%) and of a total of 568 Ebola negative patients, 117 were malaria positive (20.6%).

### External quality assessment

In two independent laboratory quality control trials the SA FEDL scored 100% in both trials [27].

### Major challenges encountered

One of the major challenges encountered during the first weeks of operation was a poorly organised system for the delivery of specimens, including: delivery of specimens very late at night, unsafe packaging resulting in specimens leaking, inappropriate primary containers (e.g. glass blood collecting tubes, syringes loaded with blood and uncapped needles wrapped in plastic bag), the delivery of specimens without patient clinical history, discrepant patient information written on primary containers versus specimen submission form, and unlabelled primary containers. Another challenge was the lack of a consistent electricity and water supply. During the first three weeks of operation, the biocontainment negative pressure chamber, refrigerators, and laboratory equipment, including PCR instruments were mostly run using the 5.5 kVA petrol generator shipped from South Africa. During the third week of operation this generator broke down due to 24 hours of continuous operation for six consecutive days, resulting in suspension of FEDL services for half a day on the 4^th^ of September 2014. Extraordinary and commendable efforts were made by local authorities to provide an emergency generator which permitted the FEDL to resume operation later that same evening. The building where the FEDL was housed was eventually connected to the national electricity power grid as well as to an emergency diesel generator provided by the Sierra Leone government. However, the provision of uninterrupted electrical power and the required maintenance for the technical infrastructure of the building often remained an unresolved issue and resulted in damage to laboratory equipment. Three PCR instruments broke down, most likely due to operation in high ambient temperature and humidity as well as problems with the power supply. Additionally the UPS unit supporting the operation of strategic laboratory equipment eventually broke down. Dysfunctional air-conditioning units made work in the biocontainment chamber, glove box and other lab areas difficult due to high temperatures and humidity and posed not only a high level of discomfort but also potentially affected human judgement resulting in errors and safety risks. Technical problems forced the laboratory to be closed for a few days in the last week of February 2015. The laboratory has since then continued to provide the Ebola diagnostic services and arrangements were made to address repairs and maintenance needs on a more regular basis. Communication via internet, including reporting of results was very time consuming and often impossible due to highly inefficient and occasionally completely dysfunctional internet and/or 3G cellular network connectivity.

### Technical improvements

The maximum daily diagnostic capacity was exceeded shortly after the establishment of the FEDL, however, the demand for EVD laboratory testing with turnaround times of less than 24 hours after receipt of specimens continued to increase. By allocating extra working time this situation was partially addressed, but such efforts were not sustainable for a longer period of time for the small SA FEDL teams. The increasing demand for Ebola infection diagnosis and the high expectation for shorter turn-around times necessitated up-scaling of the laboratory capacity and the deployment of more staff. This was eventually achieved by the provision of additional local human resources, the use of additional PCR instruments as well as technical improvements to the laboratory. These included the redesign of the PCR master mix room and the use of UV lamps in the PCR master mix room and RNA extraction room, the replacement of the rapid containment kit by a biosafety cabinet, which allowed quicker and safer addition of RNA templates to PCR master mixes, and the use of the MagMAX^™^ 96 Express automated nucleic acid extraction system which increased the speed and capacity for extraction of RNA from clinical specimens. The use of a lockable -80°C freezer improved the capacity for the secure and long-term storage of processed and tested clinical materials before shipping them to a maximum biocontainment facility (BSL-4) in Johannesburg, South Africa for further laboratory analysis and research, including the evaluation of the prototype Cepheid GeneXpert Ebola diagnostic assay [28].

### Handover of the SA FEDL to the SL MoHS

On the 17^th^ of March 2015, a meeting took place at the SL MoHS, Freetown, to discuss the protocol for the official handover of the FEDL. The meeting was attended by the Minister of Health and Sanitation, Chief Medical Officer, the Director of Hospital and Laboratory Services, and NICD staff. During this meeting the handover protocol was accepted and agreed to be fully implemented. Consequently, on the 21^st^ of March 2015 an on-site meeting was held at FEDL Freetown-Lakka to finalise the protocol and procedures for handover. On 23 March 2015 the NICD delegation visited the President of Sierra Leone, Dr Ernest Bai Koroma to report on the South African work in combating the EVD in Sierra Leone and specifically to discuss the handover of the SA FEDL to the SL MoHS. The official handover and donation of all laboratory, biosafety, and office equipment of the FEDL as well as diagnostic reagents allowing testing of at least 20,000 specimens took place on 24 March 2015. The handover meeting was attended by several representatives of the Sierra Leone MoHS, including the Deputy Minister of Health, Deputy Chief Medical Officer, the Director Hospitals and Laboratory Services, the National Manager Laboratory Services, the Deputy Manager of the National Laboratory Services, the Sierra Leonean staff trained by NICD in the operation of the laboratory, and the staff of the NICD. Documents that were reviewed, signed and issued during the handover meeting included: the Safety and Operational Manual of the FEDL, training documents and the certification of local staff in diagnostic and operational competence, equipment inventory list, reagents and consumables inventory list, laboratory specimen register, EVD investigation forms, and the agreement on FEDL capacitation by the NICD after handover to MoHS which would be on-going until the end of the epidemic. The technical support included further guidance and *ad hoc* consultation via telecommunication, the supply of reagents, and consumables and financial assistance with repairs of air conditioners and water supply systems. The FEDL was fully decontaminated and its function as EVD diagnostic laboratory terminated on 12 November 2016.

Sierra Leonean staff taught by NICD trained ten additional nationals to enhance local rapid response team capacity for epidemic-prone diseases.

## Discussion

The Western Urban and Western Rural Area where the SA FEDL operated remained a “hotspot” of the EVD epidemic in Sierra Leone for many months; from 23 May 2014 to 31 January 2015 districts in these areas, which include Freetown, had the highest number of confirmed cases (n = 3158), 39.2% of all cases nationwide [17]. At the time the first SA FEDL team arrived in Freetown delays in testing samples for EVD kept patients stranded for days in isolation wards and contributed to fears raised against seeking treatment. Severely sick patients who eventually recovered after admission to a hospital had watched Ebola virus killing others in the same room and it took days to test specimens from suspected EVD cases [16]. The nearest EVD diagnostic capacity available was at the KGH located 300 km east from Freetown (about five hours drive). At that time the laboratory was overloaded with blood samples from around the country. The delay in the laboratory confirmation of EVD suspected cases meant that patients were dying before they could be transported to a treatment centre. Within 6 days upon the arrival of the first SA FEDL team, diagnostic results were being sent to isolation wards twice a day and allowed some patients to leave the hospital on the same day that they were admitted [16]. Most results (87.6%) were issued by SA FEDL within less than 6–12 h after receiving of specimens. The test procedures only, from RNA extraction to reading RT-PCR results for each RT-PCR run (max 29 specimens per run including controls run on two Smartcyclers) took less than 4 h. However, diagnostic process concerns not only conducting an assay. Due to the high number of samples received daily and irregular delivery times specimens were batched to conduct at least two diagnostic assay runs per day: morning and afternoon runs. Much time had to be spent to safely receive and handle specimens submissions often delivered unsafely packed and/or collected in unsafe primary containers, including blood-filled syringes with needles attached, all warped in plastic shopping bags. Also much time had to be spent on qualifying samples for testing due to discrepancies of information provided in submissions (investigation) forms versus those written on primary containers. For these reasons even the most experienced laboratory could on average process only about 71% of samples on the day they arrived at the laboratory [29]. Intensification and improved coordination of outbreak control measures, including timely case finding and contact tracing, and collection of specimens resulted in significant shortening of time between onset of clinical symptoms and diagnosis, and between collection and diagnosis. Improvements of laboratory capacity enabled more rapid testing and reporting of results. In the future, field laboratories will likely utilise simpler on-site detection techniques, including point-of-care testing [28] to reduce time from sample collection to laboratory diagnosis.

Rapid and accurate laboratory confirmation of EVD cases was paramount for case management and contact tracing, and the subsequent control of the outbreak. Some of the major contributions of the SA FEDL included: (1) augmenting the local diagnostic capacity, (2) alleviating the problem of logistics that may have led to delayed testing when specimens had to be shipped to regional or international reference laboratories for testing, (3) aiding in patient management that involved resource intensive barrier nursing and isolation of suspected cases, (4) rapid molecular testing of buccal swabs from deceased persons that was essential to manage secure burials.

Training of local staff in all operations of the SA FEDL was instrumental in the long-term provision of EVD diagnostic services by this facility. It was highly beneficial for both partners, including the cost-effectiveness of laboratory operation when demand for testing decreased towards the end of the outbreak. Initially the proposal of incorporating Sierra Leonean staff in laboratory operations during the acute phase of the EVD outbreak was met with some resistance and there were doubts as to whether this could be achieved. The SA FEDL was the only facility which undertook training of national staff during the escalation stage of the EVD outbreak in the Western Urban and Western Rural Area of Sierra Leone. Most other international laboratories planned training of Sierra Leonean nationals towards the end or after the outbreak. The successful training and incorporation of national staff into all SA FEDL operations was possible because of the trust of national staff in the safety procedures and biocontainment devices used, including the high level of both primary and secondary biocontainment barriers and the use of the best available portable biocontainment devices. Having finally contained the EVD situation in March 2016 [13], Sierra Leone has maintained heightened surveillance with testing of all reported deaths and prompt investigation and testing of all suspected cases. The SA FEDL played an important role in the WHO-recommended enhanced surveillance for EVD post-outbreak until it was decommissioned in November 2016.

By the end of September 2014 there were only three field-established laboratories operating in Sierra Leone., An additional eight laboratories were established between early December and mid-January 2015 The combined effort and collaboration of these laboratories, of which six operated in the most affected Western Area of Sierra Leone, eventually not only helped to address the high demand for laboratory EVD diagnosis but also increased the total EVD diagnostic capacity in Sierra Leone. All of the field-established laboratories in Sierra Leone used real-time RT-PCR diagnostic assays [30] targeting either the viral polymerase (L) [26] or nucleocapsid (NP) and VP40 genes of EBOV [29]. However, these assays were not extensively validated in the field, mostly due to limited availability of clinical specimens in the past. Our results confirm earlier findings that buccal swabs are a useful alternative specimen source to whole blood/serum [31], and that this non-invasive specimen collection should be considered in testing unexpected community deaths. Testing of post-mortem throat swabs was also shown to be a reliable and sensitive method for EVD diagnosis and surveillance [32]. The levels of EBOV RNA detection and duration of viremia in our study were similar to other published results [33, 34]. EVD patients who have a Ct of <17 have a casa fatality rate (CFR) of 95%, and those with a Ct of >26 have a CFR of 15% [32]. It was also shown that most blood samples having Ct value > 33.7 tested negative for replicating EBOV [28]. Thus the RT-PCR Ct value could be used in discrimination between infectious and non-infectious specimens and has strong prognostic utility for aiding EVD patient management and care.

It was not always possible to retest EVD suspect patients. In this context, one had to emphasise, that a single PCR result needed to be interpreted with great caution with regards to a definitive confirmation of EVD diagnosis. Inhibitors present in the sample may have caused low or no amplification resulting in false negative results. Common inhibitors derived from red blood cells include haemoglobin and lactoferrin that may be released in haemolysed specimens. In addition molecules such as bile, CaCl_2_, EDTA, FeCl_3_ and heparin may also inhibit *Taq* polymerase activity [35]. The presence of inhibitory factors in a biological specimen is difficult to anticipate. To address some of these problems, the human betaglobin gene was used as a control for sample integrity in some of the RT-PCR protocols [29].

It is recommended that a PCR result be interpreted along with other test results where available (e.g. serology) whilst also considering the clinical history of the case. When possible, results should be confirmed on subsequent or repeat specimens. Typically, very low stringency PCR conditions are used and erroneous amplification may occur. Furthermore nucleic acid amplification methods are prone to contamination and this should always remain a concern. One of the concerns related to potentially uncertain results was associated with handling and testing of large numbers of specimens over a long time and under pressing circumstances which might contribute to contaminations. Each RT-PCR run usually consisted of specimens with very high to low concentration or no EBOV presence, thus specimens yielding high Ct values could potentially represent cross-contaminations. Consequently, these specimens were always retested using re-extracted RNA. The presence of RT-PCR inhibitors is a potential problem, but likely not very common. Most field laboratories operating in West Africa were using RT-PCR assay for the detection of EBOV L gene. There was a concern that the unprecedented number of EVD cases in West Africa (exceptional high number of EBOV person-to-person transmissions) might induce mutations which could impact the diagnostics performance of the RT-PCR. The L gene represents a much more conserved part of the EBOV genome than glycoprotein (GP) and nucleocapsid (NP) genes. One then can argue that mutations (evolution of the virus during the outbreak) would rather hamper diagnostic performance of RT-PCR targeting GP and NP than RT-PCR targeting L gene. In our recent work we demonstrated 100% correlation between RT-PCR targeting EBOV L gene and virus isolation. In addition we demonstrated that the performance of the L-gene based Taqman assay was highly comparable to an assay targeting two different genome targets [28].

Although nucleic acid sequencing may be performed to determine the molecular identity of an amplicon for confirmation, this capacity is usually not available in field-operated mobile laboratories. Due to overwhelming demand for rapid diagnosis of EVD suspected cases, and technical and staff limitations of the SA FEDL, serological testing could not be conducted. The same applied to most if not all mobile laboratories deployed in West Africa. As for most viral haemorrhagic fevers (VHFs), the non-specific presentation, especially in early stages of infection, makes it difficult to diagnose clinically. Therefore, the differential diagnosis concerns a broad array of conditions (e.g. malaria, rickettsial infections, Q fever, typhoid fever, dysentery, plague, brucellosis, leptospirosis, meningitis, other sepsis from bacterial infections, viral hepatitis, different VHFs, and non-infectious causes of disseminated intravascular coagulopathy) especially during a yet unrecognised outbreak/causative agent. Emergency laboratory response cannot provide brood spectrum differential diagnosis for suspected VHFs. However, once the outbreak is recognised, the laboratory response is mostly focused on confirmation of cases. As for all VHFs diagnostic process has to consider all available laboratory results in the context of clinical, pathological and epidemiological data.

The value of differential diagnosis was clearly demonstrated when we started testing EVD suspected cases for malaria infection towards the end of the outbreak; most of the cases turned out to be malaria positive and negative for EBOV infection.

Design, biosafety and biocontainment equipment used by international field-deployed laboratories in Africa differ from laboratory to laboratory [29–31, 36–37]. Currently there is no approved or recommended international standard for a mobile laboratory for the diagnosis of filovirus infections. Security, safety and health risks are always present in mobile missions, but can be mitigated by careful planning, mission preparation and team training [37]. However, field-deployed laboratories should not only enable rapid detection of EBOV or other highly dangerous pathogens, but their operation should only be permitted under high-security conditions with strict biosafety measures to ensure safe diagnosis of BSL-4 agents in developing and poorly- resourced countries.

One of the biocontainment features of the SA FEDL that was highly appreciated and valued was the “hot laboratory” equipped with an IsoArk and a negatively pressurised glovebox that local staff accepted as maximised and a highly efficient physical protection against biohazardous clinical materials as well as protection of the environment. As a portable, modular compact unit with all the components fitting into one robust transportation container, the IsoArk can be safely moved to a place where it is needed. One of the advantages of the IsoArk is that it is specifically designed for rapid setup, allowing the conversion of any room or space into a biologically contained area for the isolation of infected or contaminated people and materials within a day upon arrival at a deployment site, and can be used for laboratory work with potentially highly infectious substances.

The high standards of bio-safety and portability of the SA FEDL, offered a cost-effective and practical alternative for rapid deployment and operation of a high-level biocontainment facility at the scene of a formidable outbreak in a country that had little capacity for laboratory diagnosis of dangerous pathogens. The modular and portable biocontainment equipment used provided highly efficient primary and secondary biocontainment measures. Of the total of 25 operators who worked in the SA FEDL over 2 years none became infected with EBOV despite processing of large numbers highly infectious clinical specimens from EVD patients.

The location of mobile laboratories in areas where EVD was spreading uncontrollably, significantly reduced the time between the collection of biological specimens and the return of results, thus making them much more effective than centralised reference laboratories located distantly from the “hot spots” of the outbreak. The shorter the delay in obtaining a laboratory result, the earlier the confirmed cases can be managed, thus facilitating the reduction in viral transmission [31, 36–38].

The EVD epidemic in West Africa created an unprecedented challenge not only for the affected countries with weak health care systems and no previous experience with EVD, but also for the most experienced and resourceful countries, institutions and networks. It highlighted the need to maintain well organised laboratory systems and networks that can be effectively managed, including implementation of new diagnostic strategies and laboratory services in response to large-scale public health emergencies. The EVD crisis exposed systemic weaknesses and emphasised the need for better strategies to streamline the development and evaluation of new diagnostic platforms, transfer of material and specimens between countries and organisations, and more effective processes for the rapid deployment of health workers with specific laboratory expertise [39]. It also highlighted the necessity for essential reforms to better govern the global system for preventing and responding to formidable disease outbreaks [22, 40] and building citizen trust in African health systems [23].

In conclusion, the bio-safety standards and the portability of the SA FEDL, offered a cost-effective and practical alternative for the rapid deployment and operation of a field-operated high biocontainment facility. The Western Urban and Western Rural Areas of Sierra Leone, where the SA FEDL operated, remained a “hotspot” of the EVD epidemic for several months and was the only Ebola diagnostic facility to respond to the overwhelming demand for EVD diagnosis for several weeks during the initial phases of the EVD crisis in the capital city, Freetown. Rapid molecular laboratory confirmation of EVD cases was crucial for the management of EVD cases, contact tracing and secure burial practices in Sierra Leone. The deployment of the SA FEDL capacity in this country also contributed to the overall international efforts in bringing the EVD outbreak in West Africa under control. This initiative became a nucleus of rolling deployments that produced much-needed medical specialists trained in the molecular diagnosis of EVD under exceptionally trying circumstances. It also constitutes the largest Ebola outbreak response in the history of the NICD and South Africa on foreign soil. The SA FEDL teams demonstrated that it is not only possible but highly beneficial to train the national staff in the course of formidable disease outbreak and accomplished their full integration into all operational and diagnostic aspects of the laboratory. The major advantages of incorporating national staff into the SA FEDL operation included not only important contributions to the EVD diagnostic service, but also the utilisation of their knowledge of local settings, including demographics, geography and custom. This facilitated the proper receiving of specimens, accurate collection of patient data, information capture, follow-up on missing or inaccurate specimen submission forms, communication with SL MoHS, the reporting of results, and the procurement of local goods and services.

This culminated in successful hand over of the laboratory to the SL MoHS who subsequently effectively managed the laboratory and provided the required EVD diagnostic services until the country was declared free of EVD and during the post EVD outbreak enhanced surveillance.

## Supporting information

S1 Video “Hot” processing of Ebola clinical specimens, PPE and decontamination procedures in South African modular, field-operated biocontainment facility in Sierra Leone. (AVI)
